# Nomogram to predict unfavorable outcome of endovascular thrombectomy for large ischemic core

**DOI:** 10.1002/acn3.51826

**Published:** 2023-06-16

**Authors:** Nannan Han, Xiaobo Zhang, Yu Zhang, Yu Liu, Yongqin Zhang, Haojun Ma, Hanming Ge, Shilin Li, Xiao Zhang, Xudong Yan, Tengfei Li, Bin Gao, Chengxue Du, Xinchao Ji, Wenzhen Shi, Ye Tian, Mingze Chang

**Affiliations:** ^1^ Department of Neurology The Affiliated Hospital of Northwest University, Xi'an No.3 Hospital Xi'an China; ^2^ The College of Life Sciences Northwest University Xi'an China; ^3^ School of Information Science and Technology Northwest University Xi'an China; ^4^ Xi'an Key Laboratory of Cardiovascular and Cerebrovascular Diseases The Affiliated Hospital of Northwest University, Xi'an No.3 Hospital Xi'an China; ^5^ Clinical Medical Research Center The Affiliated Hospital of Northwest University, Xi'an No.3 Hospital Xi'an China

## Abstract

**Objective:**

The prognosis for patients presenting with a large ischemic core (LIC) following endovascular thrombectomy is relatively poor. This study aimed to construct and validate a nomogram for predicting 3‐month unfavorable outcome in patients with anterior circulation occlusion‐related LIC who underwent endovascular thrombectomy.

**Methods:**

A retrospective training cohort and a prospective validation cohort of patients with a large ischemic core were studied. The diffusion weighted imaging related radiomic features and pre‐thrombectomy clinical features were collected. After the selection of relevant features, a nomogram predicting modified Rankin Scale score of 3–6 as an unfavorable outcome was established. The discriminatory value of the nomogram was evaluated with a receiver operating characteristic curve.

**Results:**

A total of 140 patients (mean age 66.3 ± 13.4 years, 35% female) were included in this study, consisting of a training cohort (*n* = 95) and a validation cohort (*n* = 45). The percentage of patients with an mRS scores of 0–2 was 30%, 0–3 was 40.7%, and 32.9% were dead. Age, National Institute of Health Stroke Scale (NIHSS) score, and two radiomic features, Maximum2DDiameterColumn and Maximum2DDiameterSlice, were identified as factors associated with unfavorable outcome in the nomogram. The nomogram demonstrated an area under the curve of 0.892 (95% confidence interval [CI], 0.812–0.947) in the training dataset and 0.872 (95% CI, 0.739–0.953) in the validation dataset.

**Interpretation:**

This nomogram, which includes age, NIHSS score, Maximum2DDiameterColumn, and Maximum2DDiameterSlice, may predict the risk of unfavorable outcome in patients with LIC caused by anterior circulation occlusion.

## Introduction

Endovascular thrombectomy is a widely accepted therapeutic approach for the treatment of acute ischemic stroke caused by large vessel occlusion in the anterior circulation and recommended by established guidelines.^1^, ^2^ However, patients with large ischemic core (LIC) have typically been excluded from randomized controlled trials (RCTs),^3^, ^4^, ^5^, ^6^ particularly in the later time window of 16–24 h,^7^, ^8^ due to the potential risk of futile recanalization and intracranial hemorrhage following hyperperfusion. As a result, a subset of patients may be overlooked and miss the opportunity for endovascular therapy.

Previous non‐randomized studies have demonstrated the potential benefits of endovascular treatment in patients with LIC.^9^, ^10^, ^11^, ^12^, ^13^ The RESCUE‐Japan LIMIT (Recovery by Endovascular Salvage for Cerebral Ultra‐acute Embolism Japan Large IscheMIc core Trial) was the first RCT to show that patients with an Alberta Stroke Program Early Computed Tomographic Score (ASPECTS) value of 3–5 can benefit from endovascular thrombectomy, with a modified Rankin Scale (mRS) score of 0–2 achieved in 14% of patients in the endovascular therapy group.^14^ The subsequent multicenter RCTs SELECT2^15^ and ANGEL‐ASPECT^16^ showed that the proportion of patients with 90‐day mRS scores of 0–2 was 20–30%. Despite this evidence, patients with poor prognosis still account for a significant proportion of those with LIC following acute ischemic stroke.

In order to address this challenge, it is crucial to identify patients who are at risk of unfavorably outcomes before endovascular thrombectomy with LIC. Radiomics features (RFs), extracted from radiological imaging, may provide valuable information. In the present study, we aimed to develop and validate a nomogram that predicts unfavorable outcome in patients with LIC undergoing endovascular thrombectomy by combining pre‐thrombectomy diffusion weighted imaging (DWI) related RFs and clinical features (CFs).

## Materials and Methods

### Patients

**Figure 1:**
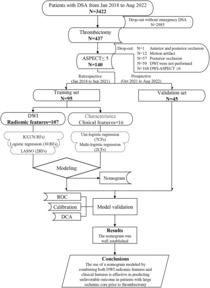
Study flowchart. DSA indicates digital subtraction angiography; CFs, clinical features; DCA, decision curve analysis; DWI, diffusion weighted imaging; ICC, intraclass correlation coefficient; LASSO, least absolute shrinkage and selection operator; RFs, radiomic features; ROC, receiver operating characteristic curve.

The current study was performed on a patient cohort (A New Parameter Derived from DSA to Evaluate Cerebral Perfusion NCT03607565) that underwent cerebral digital subtraction angiography (DSA) at a neurology department. The present study enrolled patients who were divided into two groups: (1) a retrospective training cohort and (2) a prospective validation cohort. The training cohort, consisting of patients admitted from January 2018 to September 2021, was used to identify significant RFs and CFs for the construction of a predictive model. The prospective validation cohort, comprising patients admitted from October 2021 to August 2022, was employed to evaluate the accuracy of the predictive model (Fig. 1). The study participants were selected based on the following inclusion criteria: (1) age of 18 years or older, (2) presence of large vessel occlusion in the anterior circulation (including the internal carotid artery and M1‐M2 segment of the middle cerebral artery) confirmed by DSA, (3) onset‐to‐arrival time not exceeding 24 h, and (4) pre‐thrombectomy DWI without signal change on fluid‐attenuated inversion recovery (FLAIR), suggesting recent infarction. The following exclusion criteria were also considered: (1) prior history of stroke with a mRS score of 3–5, (2) CT or MRI before thrombectomy demonstrating brain tissue swelling with midline shift, (3) known allergies (more severe than skin rash) to contrast agents, (4) evidence of acute intracranial hemorrhage in CT or MRI, (5) high risk of hemorrhage (platelet <40,000/μL), (6) DWI motion artifacts that make it difficult to accurately identify and measure the infarcted area, (7) simultaneous occlusion in both anterior and posterior circulation, and (8) DWI‐ASPECT score of ≥6 (Fig. 1).

This study was approved by the Institutional Review Board of the Affiliated Hospital of Northwest University (No. SYXSLL‐2018‐010) and the legally authorized representatives agree to undergo surgery. The supporting data for the results of this study can be made available upon request from the corresponding author. There was no involvement from any commercial entity in the design, conduct, or reporting of this research.

### Clinical data collection

The demographic and clinical characteristics of the patients were analyzed, including age, gender, medical history (previous stroke, hypertension, diabetes mellitus, hyperlipidemia, atrial fibrillation, and smoking), and severity of the ischemic stroke, which was assessed using the National Institutes of Health Stroke Scale (NIHSS) score at the emergency department. Other parameters recorded included the site of the occlusion, the time from stroke onset to DWI imaging, the time from DWI imaging to DSA imaging, presence of wake‐up stroke (the estimated time point was calculated as the midpoint between the last normal time and the time when the symptoms were detected^17^), and administration of intravenous recombinant tissue plasminogen activator (rt‐PA) or recombinant human TNK tissue‐type plasminogen activator (TNK).

For the purpose of comparing differences between the training and validation sets, additional parameters were recorded, including DWI‐ASPECT score, infarction volume, DSA duration, cause of stroke, thrombectomy procedure, reperfusion status according to the Thrombolysis in the Cerebral Infarction (TICI) grading system^18^ (2b‐3 as an indicator of successful reperfusion), and the occurrence of any intracranial hemorrhage^19^ within 48 h. The favorable outcome was defined as a mRS score of 0–2, fair outcome as mRS 0–3, and death (mRS score of 6) was evaluated 90 ± 7 days after thrombectomy through either a telephone or a face‐to‐face interview with the patient or their relatives, conducted by neurologists.

### DWI‐ASPECT assessment and radiomic features extraction

The DWI scans were acquired using a PHILIPS Ingenia 3.0T scanner with the following parameters: repetition time of 2506 ms, echo time of 68 ms, field of view of  230 × 230 mm, image matrix of 152 × 102 pixels, b‐values of 0 and 1000 s/mm,^2^ section thickness of 5.0 mm, and interslice gap of 1.0 mm. The DWI data were automatically uploaded to the Picture Archiving and Communication System (PACS) and then downloaded in DICOM format and submitted to the laboratory for evaluation by two independent readers. Two neurologists manually segmented all areas of hyperintense signals in DWI using 3D Slicer (version 4.11.2).^20^ The DWI‐ASPECT score was determined by only two slices, one at the level of the thalamus and basal ganglia, and one just rostral to the ganglionic structures,^21^ in order to prevent the high signal in other slices from reducing the DWI‐ASPECT score. The hyperintense signals in the two slices were scored as 0 (abnormal) or 1 (normal), in accordance with the CT‐ASPECT score regions on DWI. The neurologists were blinded to the results of the endovascular procedure and clinical outcome.

The RFs^22^ were automatically extracted from the segmented hyperintense signal region using Pyradiomics (version 3.0.1). A total of 107 radiomic features were extracted from the DWI data, including First Order Statistics (18 features), Shape‐based (3D) (14 features), Gray Level Cooccurrence Matrix (24 features), Gray Level Run Length Matrix (16 features), Gray Level Size Zone Matrix (16 features), Neighboring Gray Tone Difference Matrix (5 features), and Gray Level Dependence Matrix (14 features).

### Statistical analysis

For the analysis of clinical features (CFs), quantitative variables were presented as either mean ± standard deviation (SD) or median with interquartile range (IQR). The normality of data distributions was determined by evaluating histograms and using the Shapiro–Wilk test, with values of *P* > 0.05 considered indicative of a normal distribution. Bivariate comparisons between CFs were conducted using either the chi‐square test (with Fisher's exact test applied when the expected cell frequency was less than 5) for categorical variables, or the Student's t‐test (or Mann–Whitney U‐test) for quantitative variables. In the univariate logistic regression analysis, factors affecting the unfavorable outcome (*P* < 01) were further evaluated in a multivariable logistic regression analysis, using a backward‐selection method. Clinical features with a *P* < 0.05 in the multivariable analysis were considered statistically significant.

For the analysis of RFs, a quality control assessment was performed using the intraclass correlation coefficient (ICC), and those with ICC values less than 0.75 were dropped. The remaining RFs were selected using a least absolute shrinkage and selection operator (LASSO), based on the results of univariate logistic regression (*P* < 0.1), in the training cohort. (Fig. 1).

The nomogram was developed using the selected CFs and RFs, and its discriminative performance was evaluated using the time depended external validation dataset. The receiver operating characteristic curve (ROC) with area under the curve (AUC) was used to measure the performance of the nomogram, while calibration plots were used to describe the degree of fit between the actual and nomogram‐predicted unfavorable outcome. The validity of the nomogram was further evaluated using the decision curve analysis (DCA).^23^ The ICC was performed using the Python 3.9.7 and Pinguin 0.5.2 package, the ROC was performed using MedCalc 19.2.0, and the RFs were extracted using the Pyradiomics 3.0.1, Scikit‐learn 0.24.2, Pandas 1.3.4, and Numpy 1.20.3 packages. Additional statistical analysis was performed using SPSS (version 26.0, IBM, NY, USA) and R statistical software (version 4.2.1, R Foundation for Statistical Computing, Vienna, Austria).

## Results

The process of patient inclusion in the study is shown in Figure 1. A total of 3422 patients underwent DSA, out of which 140 patients met the inclusion and exclusion criteria. The retrospective training cohort included 95 patients, while the prospective validation cohort included 45 patients. A total of 3282 did not meet the inclusion and exclusion criteria, 2985 of did not receive emergency DSA, 1 experienced simultaneous occlusion in both the anterior and posterior circulations, 12 had poor‐quality DWI images, 59 lacked DWI scans, and 168 patients had DWI‐ASPECT scores of 6 or higher.

The mean age of the patients with DWI‐ASPECT score 0–5 was 66.3 ± 13.4 years, with 49 (35.0%) being female. The mean NIHSS score at admission was 16.3 ± 6.2, and the median DWI‐ASPECT score was 4. The occlusion sites in the study population were primarily located in the internal carotid artery (46.4%), followed by the M1 segment of the middle cerebral artery (MCA) (42.9%), and the M2 segment of the MCA (10.7%). Atrial fibrillation was detected in 42.1% of the patients, and 24.3% of the patients received rt‐PA/TNK treatment. In terms of functional outcome, 30.0% of the patients had a mRS score of 0–2, 40.7% had a fair outcome as a mRS score of 0–3, and 32.9% had a death as a mRS score of 6. The majority (90.0%) of the patients had a TICI reperfusion grade of 2b or higher, and the incidence of all types of hemorrhage was 48.6% (Table 1).

**Table 1:** Demographics and characteristics of the training and validation cohorts.

Variable	All patients (*N* = 140)	Training set (*N* = 95)	Validation set (*N* = 45)	*P* value
mRS 0–2, *n* (%)	42 (30.0)	27 (28.4)	15 (33.3)	0.554
mRS 0–3, *n* (%)	57 (40.7)	36 (37.9)	21 (46.7)	0.324
mRS 6, *n* (%)	46 (32.9)	33 (34.7)	13 (28.9)	0.491
Age, years, mean ± SD	66.3 ± 13.4	67.0 ± 12.9	64.7 ± 14.5	0.330
Male sex, *n* (%)	91 (65.0)	61 (64.2)	30 (66.7)	0.776
Previous stroke, *n* (%)	30 (21.4)	21 (22.1)	9 (20.0)	0.777
Hypertension, *n* (%)	86 (61.4)	58 (61.1)	28 (62.2)	0.894
Diabetes mellitus, *n* (%)	30 (21.4)	18 (18.9)	12 (26.7)	0.299
Hyperlipidemia, *n* (%)	26 (18.6)	15 (15.8)	11 (24.4)	0.219
Atrial fibrillation, *n* (%)	59 (42.1)	45 (47.4)	14 (31.1)	0.069
Smoking, *n* (%)	50 (35.7)	36 (37.9)	14 (31.1)	0.434
NIHSS score, mean ± SD	16.3 ± 6.2	16.4 ± 6.7	16.3 ± 5.2	0.967
Occlusion site, *n* (%)				
Internal carotid artery	65 (46.4)	40 (42.1)	25(55.6)	0.136
M1 segment of MCA	60 (42.9)	43 (45.3)	17 (37.8)	0.403
M2 segment of MCA	15 (10.7)	12 (12.6)	3 (6.7)	0.439
DWI‐ASPECTS				
Median value (IQR)	4 (3, 5)	4 (3,5)	4 (3,5)	
0 *n* (%)	1 (0.7)	0 (0.0)	1 (2.2)	0.701
2 *n* (%)	13 (9.3)	8 (8.4)	5 (11.1)	0.841
3 *n* (%)	40 (28.6)	30 (31.6)	10 (22.2)	0.252
4 *n* (%)	30 (21.4)	22 (23.2)	8 (17.8)	0.469
5 *n* (%)	56 (40.0)	35 (36.8)	21 (46.7)	0.268
Infarction volume, mm^3^, mean ± SD	83.54 ± 72.76	92.21 ± 71.76	65.26 ± 72.24	0.004
Time from onset to DWI, min (IQR)	321.5 (183.3, 505.0)	305.0 (177.0, 488.0)	395 (194.0, 516.5)	0.165
Time from DWI to DSA, min (IQR)	78 (61.3, 99.0)	78 (62.0, 101.0)	80.0 (58.5, 96.5)	0.672
DSA duration, min (IQR)	109.5 (81.5, 156.5)	116.0 (87.0, 162.0)	94.0 (69.5, 138.5)	0.103
Woken up stroke, *n*. (%)	46 (32.9)	30 (31.6)	16 (35.6)	0.640
Intravenous rt‐PA or TNK	34 (24.3)	20 (21.1)	14 (31.1)	0.195
Cause of stroke, *n* (%)				
Embolic	86 (61.4)	64 (67.4)	22 (48.9)	0.036
Atherosclerotic	47 (33.6)	26 (27.4)	21 (46.7)	0.024
Others	7 (5.0)	5 (5.3)	2 (4.4)	1.000
Procedural modes, *n* (%)				
ADAPT only	52 (37.1)	32 (33.7)	20 (44.4)	0.218
Stent retriever	46 (32.9)	40 (42.1)	6 (13.3)	0.001
Balloon or/and stenting	42 (30.0)	23 (24.2)	19 (42.2)	0.030
Reperfusion, *n* (%)	126 (90.0)	83 (87.4)	43 (95.6)	0.228
Hemorrhage, *n* (%)	68 (48.6)	46 (48.4)	22 (48.9)	0.959

ADAPT, a direction aspiration first‐pass technology; DSA, digital subtraction angiography; DWI, diffusion weighted imaging; IQR, interquartile range; MCA, middle cerebral artery; mRS, modified Rankin Scale; NIHSS, national institute of the health stroke scale; rt‐PA, intravenous recombinant tissue plasminogen activator; SD, standard deviation; TNK, recombinant human TNK tissue‐type plasminogen activator.

The baseline characteristics of the training and validation cohorts are presented in Table 1. There were no statistically significant differences in the proportion of favorable outcome, defined as mRS score of 0–2, with 28.4% in the training cohort and 33.3% in the validation cohort (*P* = 0.554). However, the mean infarction volume in the training cohort was larger (92.21mm^3^ vs 65.26 mm^3^, *P* = 0.004), with a higher proportion of embolic occlusions (67.4% vs. 48.9%, *P* = 0.036) and a lower proportion of patients who received rescue treatments such as emergency balloon or stenting (24.2% vs. 42.4%, *P* = 0.030).

The results of the logistic regression analysis for unfavorable outcome (mRS 3–6) at 90 days in the training cohort are presented in Table 2. The univariate analysis indicated that age, gender, hypertension, atrial fibrillation, current smoking, NIHSS score, and time from stroke onset to DWI imaging were potential predictors of unfavorable outcome (*P* < 0.1). The multivariate analysis revealed that age (Odds Ratio [OR], 1.085; 95% confidence interval [CI], 1.030–1.142; *P* = 0.002) and NIHSS score (OR, 1.287; 95% CI, 1.130–1.446; *P* < 0.001) was significant predictors of unfavorable outcome. Additionally, 107 RFs were extracted from the segmented regions of two readers, and 76 cases with an ICC greater than 0.75 were selected for further analysis. The results of the univariate logistic regression showed that 30 cases had a significant relationship with the outcome (*P* < 0.1). Subsequently, LASSO analysis was performed, and two RFs, Maximum2DDiameterColumn and Maximum2DDiameterSlice, were selected as significant predictors of the unfavorable outcome (Figure S1). Maximum2DDiameterColumn and Maximum2DDiameterSlice are two RFs that exhibit a high degree of correlation with the infarct volume. Specifically, Maximum2DDiameterColumn is defined as the largest pairwise Euclidean distance between the vertices of the infarct surface mesh in the row‐column plane, which is generally aligned with the axial plane. On the contrary, Maximum2DDiameterSlice is defined as the largest pairwise Euclidean distance between the vertices of the infarct surface mesh in the row‐slice plane, which typically corresponds to the coronal plane.

**Table 2:** Logistic regression for the risk factors associated with unfunctional outcome in the training cohort.

Variable	Unadjusted OR (95% CI)	*P* value	Adjusted OR (95% CI)	*P* value
Age, years	1.084 (1.038–1.133)	<0.001	1.085 (1.030–1.142)	0.002
Sex	3.272 (1.107–9.668)	0.032	0.639 (0.120–3.386)	0.598
Previous stroke	1.917 (0.580–6.334)	0.286		
Hypertension	2.614 (1.048–6.515)	0.039	2.401 (0.717–8.045)	0.156
Diabetes mellitus	1.040 (0.331–3.264)	0.946		
Hyperlipidemia	1.110 (0.320–3.844)	0.870		
Atrial fibrillation	3.619 (1.352–9.690)	0.010	0.483 (0.104–2.241)	0.353
Smoking	0.228 (0.089–0.586)	0.002	0.385 (0.112–1.324)	0.130
NIHSS score	1.287 (1.146–1.445)	<0.001	1.287 (1.130–1.446)	<0.001
Occlusion site				
Internal carotid artery	2.111 (0.814–5.478)	0.125		
M1 segment of MCA	0.560 (0.228–1.377)	0.207		
M2 segment of MCA	0.767 (0.210–2.793)	0.687		
Time from onset to DWI, min	0.998 (0.996–1.000)	0.015	0.999 (0.997–1.002)	0.577
Time from DWI to DSA, min	1.002 (0.991–1.014)	0.662		
Woken up stroke	1.460 (0.539–3.956)	0.456		
Intravenous rt‐PA or TNK	2.667 (0.713–9.980)	0.145		

ADAPT, a direction aspiration first‐pass technology; CI, confidence interval; DSA, digital subtraction angiography; DWI, diffusion weighted imaging; MCA, middle cerebral artery; NIHSS, national institute of the health stroke scale; OR, odd ratio; rt‐PA, intravenous recombinant tissue plasminogen activator; TNK, recombinant human TNK tissue‐type plasminogen activator.

**Figure 2:**
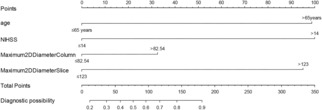
Nomogram for predicting the unfavorable outcome (modified Rankin Scale 3–6). Points were assigned for age, NIHSS, Maximum2DDiameterColumn, and Maximum2DDiameterSlice by drawing a line upward from the corresponding values. The total points are calculated as each individual score of the 4 variables included in the nomogram. For example, a 74‐year‐old (score of 99 points due to being over 65) patient with an NIHSS score of 18 (score of 100 points due to being over 14), a Maximum2DDiameterColumn of 98.41 mm (score of 32 points due to being over 82.54), and a Maximum2DDiameterSlice of 113 mm (score of 0 points due to being below 123) was assessed. The patient had a Total Points score of 231, corresponding to a diagnostic possibility of more than 90%, suggesting an unfavorable outcome. NIHSS indicates national institute of the health stroke scale.

The two CFs and RFs were dichotomized based on the ROC curve. Age and NIHSS score were dichotomized using the maximum Youden index, with the cutoff points being ≤65 years and >65 years, and ≤14 and >14, respectively. Given the limitations of the sample size, this study has employed cutoff points to dichotomize Maximum2DDiameterColumn and Maximum2DDiameterSlice to prevent overfitting. The Maximum2DDiameterColumn and Maximum2DDiameterSlice were dichotomized at cutoff points of ≤82.54 mm and >82.54 mm, and ≤123 mm and >123 mm, respectively (Figure S2). The results indicated that higher total points, as calculated by summing the assigned number of points for each predictor in the nomogram, corresponded to an increased risk of unfavorable outcome (Fig. 2).

**Figure 3:**
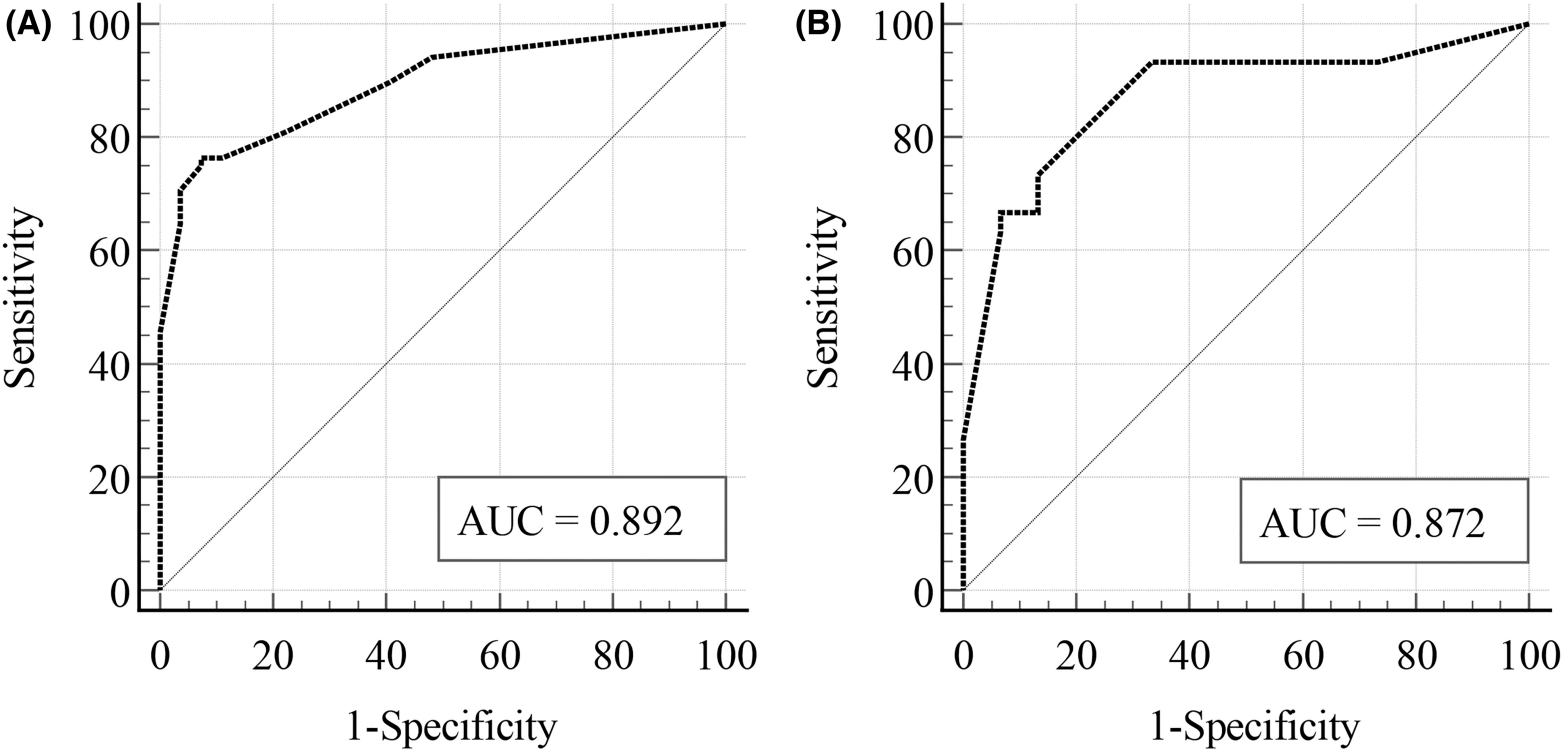
Receiver operating characteristic (ROC) curve of the nomogram for predicting 3‐month unfavorable outcomes of stroke with large ischemic core treated with mechanical thrombectomy in training cohort (A), and validation cohort (B). AUC indicates area under the curve.

**Figure 4:**
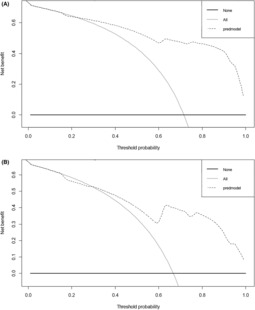
Decision curve analysis (DCA) of the nomogram was performed in both the training cohort (A) and validation cohort (B). The gray line represents the net benefit of the strategy of treating all patients, while the black line indicates the net benefit of the strategy of treating no patients. The dotted line illustrates the performance of the nomogram, which was developed to assess the probability of unfavorable outcome at 90 days post‐thrombectomy for a given patient. In the present study, the reference risk was calculated by assuming that all patients need further treatment for preventing unfavorable outcome, whereas zero net benefit was defined as no patients needing further therapy. The threshold probability is when the expected benefit of further therapy is equal to the expected benefit of avoiding further therapy. For a threshold probability >23% in the training cohort and >29% in the validation cohort, application of the nomogram would add net benefit compared to either the treat‐all strategy or the treat‐none strategy.

The performance of the nomogram was assessed by the AUC of ROC, which was found to be 0.892 (95% CI, 0.812–0.947) in the training dataset and 0.872 (95% CI, 0.739–0.953) in the validation dataset, indicating good predictive power (Fig. 3A and B). The calibration plot was used to compare the prediction of unfavorable outcome made by the nomogram with actual observations, and it showed good accuracy in both the training and validation datasets (Figure S3).The results of the decision curve analysis showed that when the threshold probabilities exceed 23% in the training cohort and 29% in the validation cohort, utilizing the nomogram for prediction of 90‐day unfavorable outcome results in a greater net benefit compared to the “treat all” or “treat none” strategies, suggesting the utility of the nomogram (Fig. 4A and B).

## Discussion

In this study, 30% of patients had favorable outcomes, approximately 40% had a fair outcome, and nearly one‐third of patients died following thrombectomy for patients with LIC. To predict the 3‐month unfavorable outcome, a nomogram was developed based on the variables of age, NIHSS score, and Maximum2DDiameterColumn and Maximum2DDiameter Slice. The performance of the nomogram in terms of discrimination and calibration was shown to be satisfactory in the training cohort and was further confirmed in a time‐dependent external validation cohort. The nomogram using RFs in combination with preoperative NIHSS and age provides a way to predict the prognosis of individual patients prior to thrombectomy.

In previous studies, the proportion of good outcomes in patients with large infarct cores who underwent endovascular thrombectomy ranged from 8% to 52%.^9^, ^10^, ^11^, ^12^, ^13^, ^24^ The reasons for this phenomenon are numerous: (1). In early studies, the proportion of successful recanalization was relatively low,^12^, ^13^ whereas in recent studies it has reached 83–86%.^9^, ^14^ (2). The etiology of large vessel occlusion varies due to ethnicity. In Asian populations, intracranial atherosclerotic occlusions are more prevalent^25^ and exhibit a prolonged compensatory establishment period, which may result in fluctuating prognostic outcomes. (3). The most critical issue is that various studies have diverse definitions of a large ischemic core. However, how much is too much have no consistent standard. The main inclusion criteria for a large infarct core were as follows: (1). Different studies have used varying cutoffs to define a large ischemic core. Specifically, the ASPECT score of ≤5 may be based on ESCAPE^4^ and SWIFT PRIME^6^ trials, whereas alternative studies have adopted volumetric measurements of infarct size, employing thresholds greater than 50 or 70 mL as their criteria, potentially stemming from the DAWN^7^ or Diffuse‐3^8^ trial. (2). Different studies have divergent definitions of infarction, with some using ADC <620 × 10^6^ mm^2^/s,^10^, ^24^ and some using the time to maximum (Tmax) delay ≥3 s and relative cerebral blood flow ≤30% calculated by software such as MIStar.^9^

The inclusion criteria for a large infarct core in the ongoing RCT study TENSION (NCT03094715) include an NCCT or DWI ASPECTS score of 3–5, which is similar to the criteria used in the published RESCUE‐Japan LIMIT study.^14^ The ongoing RCT study LASTE (NCT03811769) uses either NCCT or DWI with ASPECTS scores ranging from 0 to 5 as the imaging inclusion criteria. Another RCT study, TESLA (NCT03805308), utilizes NCCT ASPECTS scores ranging from 2 to 5 as the imaging inclusion criterion. The SELECT2 study^15^ included 178 patients in the endovascular therapy group, of whom 20% achieved functional independence (mRS score 0–2), which was statistically different compared to 7% in the medical care group. The ANGEL‐ASPECT study^16^ included 230 patients in the endovascular therapy group, of whom 30% achieved functional independence, compared to 11.6% in the medical care group. Additionally, the endovascular therapy group mortality rate was 38% for SELECT2 and 21.7% for ANGEL‐ASPECT. These two studies indicate the potential existence of different prognostic profiles for different definitions of large ischemic core. The uniformity of CT‐ASPECT scores among neurologists is poor.^26^ Therefore, in this study, a DWI‐ASPECT score of ≤5 was employed as the imaging inclusion criterion for LIC. This choice was based on the superior discrimination and uniformity of the DWI‐ASPECT score compared to the CT‐ASPECT score. DWI is better at visualizing early cerebral infarct lesions and, as a result, may produce DWI‐ASPECT scores that are lower than CT‐ASPECT scores,^27^ potentially leading to an overestimation of core infarct volume. In addition, the infarct volume may vary greatly for each DWI‐ASPECT score,^28^ and the finding was observed in this study.

In our study, the proportion of patients with a mRS score of 0–2 was 30%, which was higher than the 14% reported in the rescue study in Japan. This discrepancy is likely due to the inclusion of a higher proportion of patients with low DWI‐ASPECT scores and small infarct sizes in our study population. Additionally, our study population showed that 33.6% of the cases of AIS were caused by atherosclerotic occlusions. The mean infarct volume in this patient subgroup was 36.3 mm^3^, and the proportion of patients who achieved functional independence was 44.7% (21/47). Importantly, our observations indicate that a low DWI‐ASPECT score may correspond to a larger area of hypoperfusion in cerebral blood flow. If the infarct volume is large, it may indicate a deficit mismatch, while a small infarct volume may suggest a favorable mismatch and a better prognosis following endovascular thrombectomy. The radiomic features Maximum2DDiameterColumn and Maximum2DDiameterSlice were associated with infarct volume. This supports the notion that the volume of the large infarct core is a significant factor affecting patient prognosis.

The four‐variable model exhibits an AUC value of 0.892 in predicting unfavorable outcome, which is significantly superior (*P* < 0.001) to the infarct volume prediction model with an AUC of 0.639. The combined model of age and NIHSS demonstrates an AUC of 0.856 in predicting unfavorable outcome, which is inferior (*P* = 0.039) to the four‐variable combined model. This model was developed to assist neurologists in identifying patients with an unfavorable outcome before thrombectomy. In cases of anterior circulation large vessel occlusion with a DWI‐ASPECT <5, the likelihood of an unfavorable outcome can be estimated based on four variables: age, NIHSS score, Maximum2DDiameterColumn, and Maximum2DDiameterSlice. The Maximum2DDiameterColumn is related to the axial view of DWI, while the Maximum2DDiameterSlice is related to the coronal view of DWI. The Pearson correlation coefficient was calculated for Maximum2DDiameterSlice and infarct volume resulting in *r* = 0.653 (*P* < 0.001), and for Maximum2DDiameterColumn and infarct volume yielding *r* = 0.717 (*P* < 0.001). Nevertheless, the employment of this model is significantly hampered by the prevalent utilization of CT for assessing stroke in most centers. In parallel, MRI has gained substantial popularity for pre‐thrombectomy evaluation, as evidenced by the Rescue‐Japan study^14^ reporting its utilization in 88% and 87% of the endovascular therapy and medical care groups, respectively. We are concerned that although multiple large RCTs have demonstrated the efficacy of thrombectomy for large infarct core in enhancing the proportion of patients who achieve functional independence, indiscriminately expanding the indications for this procedure may harm certain patients with poor outcomes. In the future, we aim to utilize continuous variables (rather than transformed dichotomous variables) for each variable as the sample size expands, to develop models that can be integrated into the PACS workstation for real‐time prognostication of patient outcomes. Consequently, this model has the potential to optimize therapeutic decision‐making.

## Limitations

This study has some limitations. First, it is a single‐center, non‐randomized study. The nature of the analyses has inherent limitations; however, the advantage of the single‐center design is that it ensures consistency of data and treatment strategies for patient management. Second, the sample size of this study is relatively small, which may limit its statistical power. The study only considers the pre‐thrombectomy period to reduce the number of variables and focus on predicting the unfavorable outcome of specific patients before thrombectomy. The data were dichotomized to develop the nomogram, which increases the simplicity of its clinical application while reducing overfitting. Third, only first order statistics were extracted within the region of interest, as the application of filters to extract higher order features was omitted to reduce overfitting and increase clinical interpretability. Fourth, the manual delineation of infarct areas is time‐consuming and can be challenging to implement in an emergency workflow. Utilizing artificial intelligence to identify and extract features automatically may provide a solution to this issue. More work is required to determine the feasibility of a RFs‐based model for clinical practice. In the interim, the utilization of NIHSS and age for an approximate prognosis estimate in emergency settings is a plausible option. Fifth, perfusion images from MR imaging were not considered in this study. Low DWI‐ASPECT scores often indicate large perfusion deficits, and mismatches within each ASPECT region are further investigated as surrogates for DWI‐ASPECT to predict patient prognosis. Finally, long‐term follow‐up more than 90 days were missing is a further limitation.

## Conclusion

Generally, the nomogram, which is composed of age, NIHSS score, Maximum2DDiameterColumn, and Maximum2DDiameterSlice, may predict the risk of an unfavorable outcome in patients with a LIC who are treated with endovascular thrombectomy. Further studies are required to validate the effectiveness of this nomogram in other patient populations.

## Conflict of Interest

The authors declare that the research was conducted in the absence of any commercial or financial relationships that could be construed as a potential conflict of interest.

## Funding Information

This study was partially supported by grants from Natural Science Basic Research Project of Shaanxi Province (2022JM‐452).

## Author Contributions

NNH conceived and collected the data, and wrote the manuscript. XBZ, YZ, and YL performed the data analysis. HJM, HMG, SLL, XZ, XDY, TFL, BG, CXD, XCJ, WZS, and YT involved in manuscript review and revision. MZC critically revised the report and accepted full responsible for the overall content. All authors contributed to the article and approved the submitted version.

## Supporting information


**Figure S1.** The process of selecting radiomic features utilizing the Least Absolute Shrinkage and Selection Operator (LASSO) binary logistic regression model is presented. (A) illustrates the LASSO coefficient profiles for the 30 radiomic features, plotted against the log (λ) sequence. (B) shows the tuning parameter (λ) selection process in the LASSO model, which was conducted through 4‐fold cross‐validation with minimum criteria. The plot displays the area under the receiver operating characteristic curve was plotted versus log (λ), with dotted vertical lines indicating the optimal value chosen according to the minimum criteria and the 1 standard error of the minimum criteria.

**Figure S2.** The two clinical features and two radiomics feature were dichotomized based on the receiver operating characteristic curve. Age (A) and NIHSS score (B) were dichotomized using the maximum Youden index, with the cutoff points being ≤65 years and >65 years, and ≤14 and >14, respectively. The Maximum2DDiameterColumn (C) and Maximum2DDiameterSlice (D) were dichotomized at cutoff points of ≤82.54 and >82.54, and ≤123 and >123, respectively.

**Figure S3.** The calibration of the nomogram was evaluated in both the training cohort (A) and validation cohort (B). The reference line, where an ideal nomogram would lie, is depicted as a gray bold line. The solid line represents the correction for any bias in the nomogram, while the dotted line represents the performance of the nomogram.
