# Supplementary figures and images for: Nomogram to predict unfavorable outcome of endovascular thrombectomy for large ischemic core
Source: Ann Clin Transl Neurol. 2023 Jun 16;10(8):1353–64. doi: 10.1002/acn3.51826 (PMC10424651; doi:10.1002/acn3.51826)

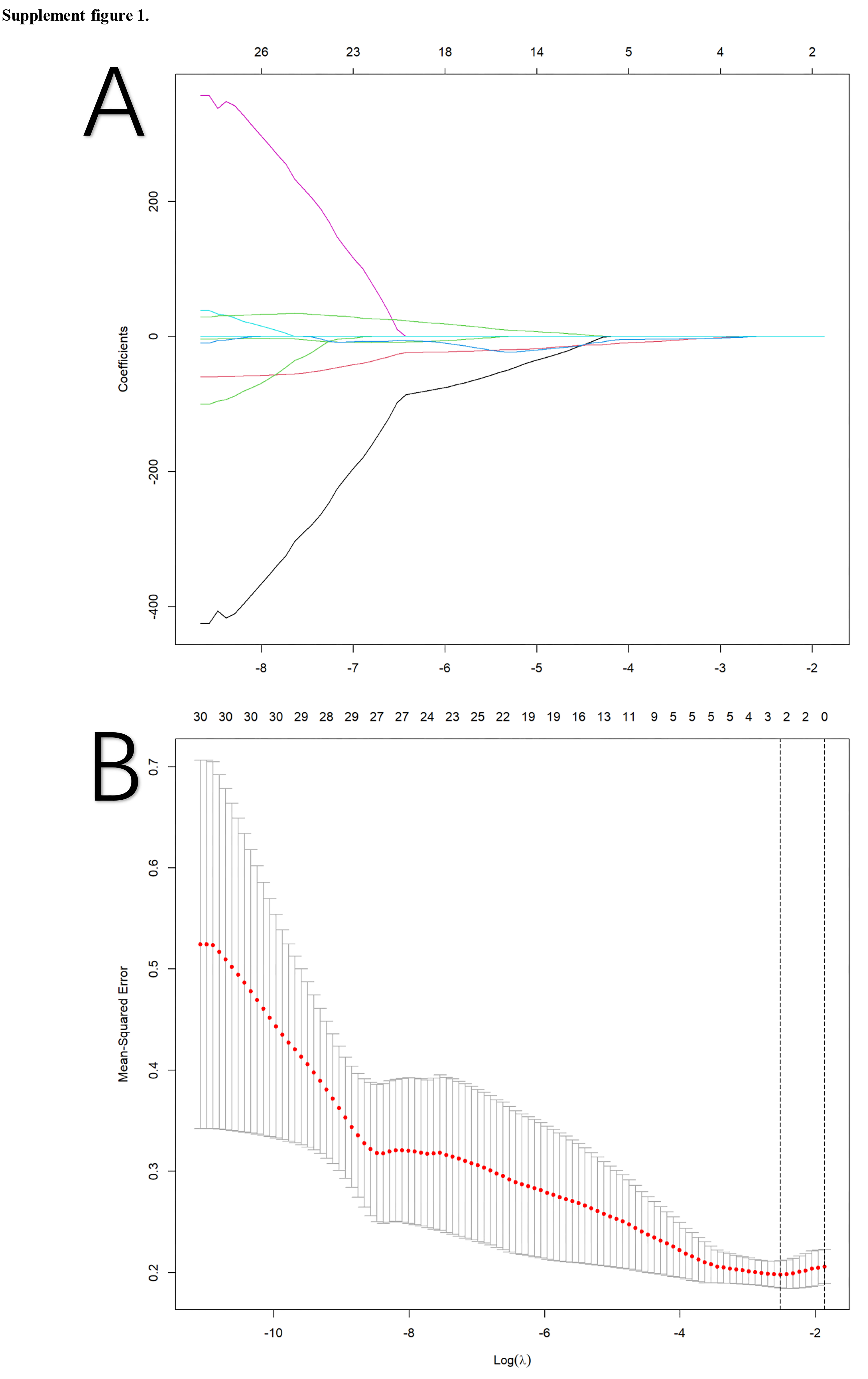

Supplement: Supplementary file 1 — Figure S1. The process of selecting radiomic features utilizing the Least Absolute Shrinkage and Selection Operator (LASSO) binary logistic regression model is presented. (A) illustrates the LASSO coefficient profiles for the 30 radiomic features, plotted against the log (λ) sequence. (B) shows the tuning parameter (λ) selection process in the LASSO model, which was conducted through 4‐fold cross‐validation with minimum criteria. The plot displays the area under the receiver operating characteristic curve was plotted versus log (λ), with dotted vertical lines indicating the optimal value chosen according to the minimum criteria and the 1 standard error of the minimum criteria. [file ACN3-10-1353-s002.png]

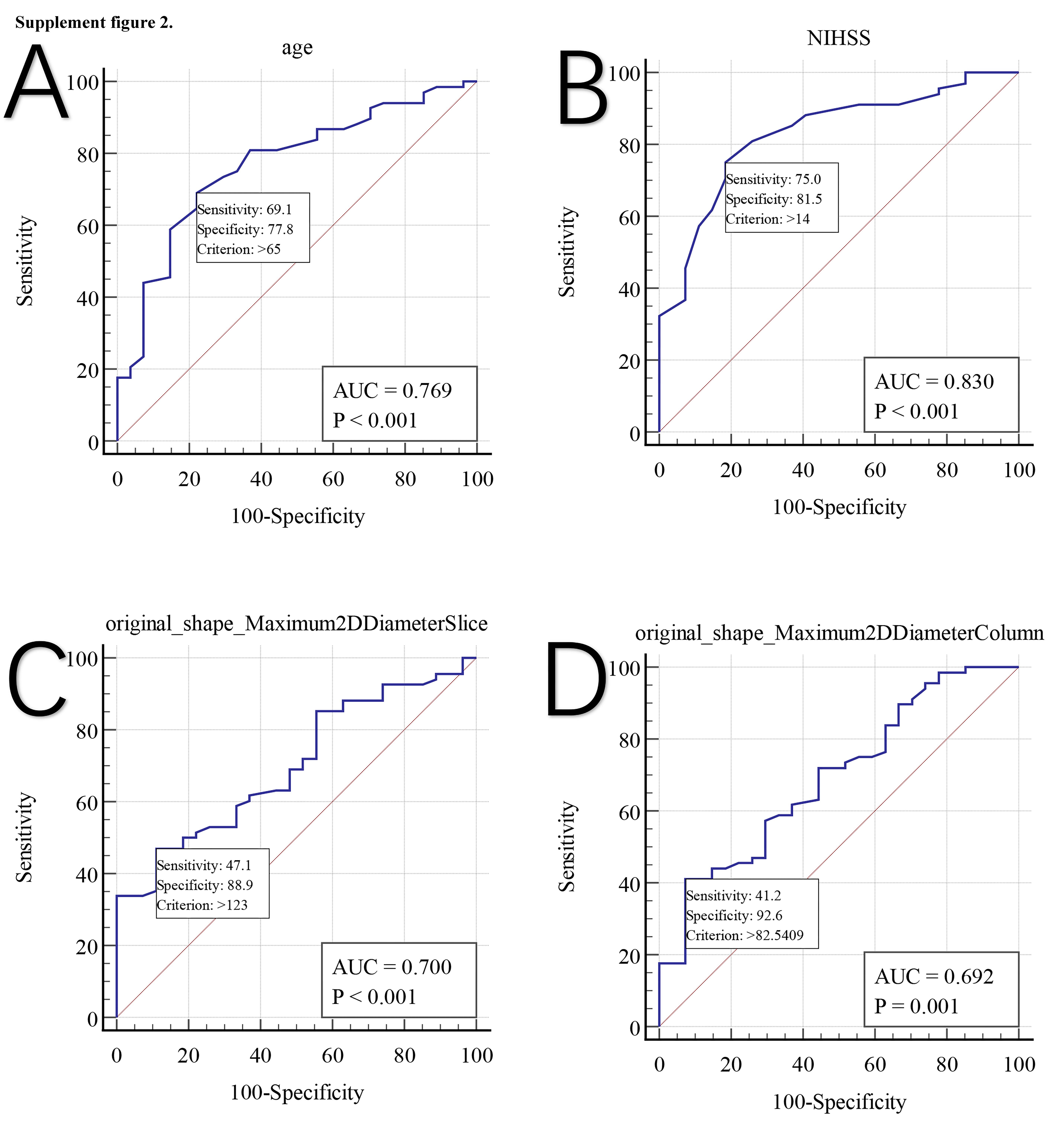

Supplement: Supplementary file 2 — Figure S2. The two clinical features and two radiomics feature were dichotomized based on the receiver operating characteristic curve. Age (A) and NIHSS score (B) were dichotomized using the maximum Youden index, with the cutoff points being ≤65 years and >65 years, and ≤14 and >14, respectively. The Maximum2DDiameterColumn (C) and Maximum2DDiameterSlice (D) were dichotomized at cutoff points of ≤82.54 and >82.54, and ≤123 and >123, respectively. [file ACN3-10-1353-s001.png]

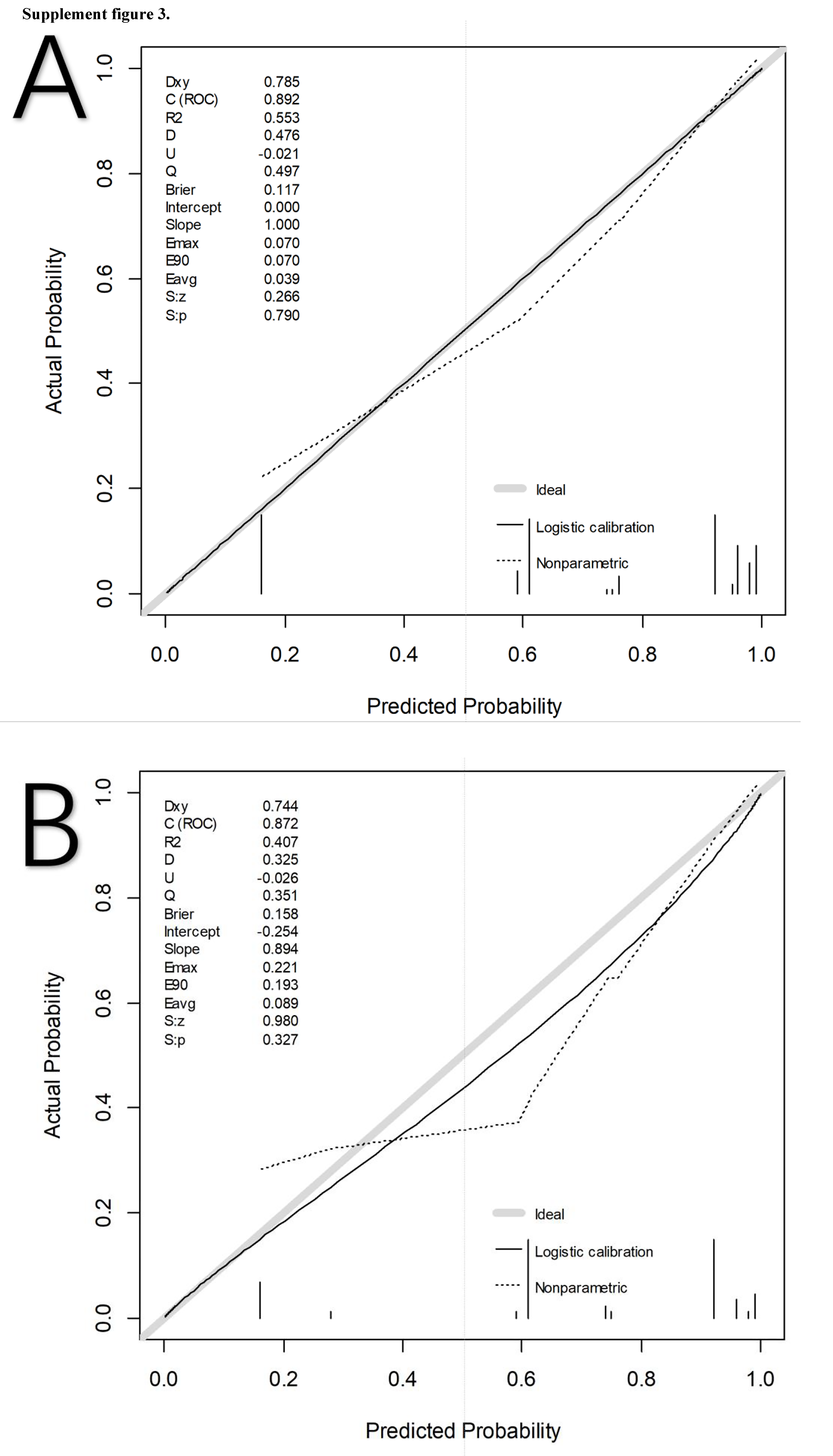

Supplement: Supplementary file 3 — Figure S3. The calibration of the nomogram was evaluated in both the training cohort (A) and validation cohort (B). The reference line, where an ideal nomogram would lie, is depicted as a gray bold line. The solid line represents the correction for any bias in the nomogram, while the dotted line represents the performance of the nomogram. [file ACN3-10-1353-s003.png]
